# Evidence-based classification in golf for athletes with a vision impairment: A Delphi study

**DOI:** 10.1111/opo.13049

**Published:** 2022-09-21

**Authors:** Niall J. Hynes, Eldre W. Beukes, Roger Hawkes, Howard A. Bennett, Christian Hamilton, Prakash Jayabalan, Peter M. Allen

**Affiliations:** 1 Centre for Vision across the Life Span, School of Applied Sciences, University of Huddersfield, Huddersfield, UK; 2 Vision and Hearing Sciences Research Centre, School of Psychology and Sports Sciences, Anglia Ruskin University, Cambridge, UK; 3 University College London, London, UK; 4 Durham University, Durham, UK; 5 Golf Australia, Australian Golf Centre, Cheltenham, Victoria Australia; 6 Shirley Ryan AbilityLab, Chicago, Illinois USA; 7 Northwestern Feinberg School of Medicine, Chicago, Illinois USA

**Keywords:** golf, low vision, sports vision, vision impairment

## Abstract

Vision-impaired (VI) golf is a global para-sport currently played under several different classification systems under different bodies. This study aimed to gather expert opinion to determine whether the current classification systems are fit for the purpose intended and to identify any particular issues where VI impacts the game of golf for the disabled (G4D). A panel of 20 participants with expertise in G4D took part in a three-round Delphi study. The panel agreed that the current classification system(s) for VI golf did not or only partially fulfilled the aim to minimise the impact of VI on the outcome of competition and that there should be one, internationally recognised, classification system. It was agreed that other metrics of VI, in addition to the measurement of visual acuity (VA), need to be considered. Intentional misrepresentation of VI was identified as a cause for concern. The panel agreed that the current classification system does not fully achieve its purpose. Any changes that are made to these classification systems need to be evidence based specific to VI golf. Further research is required to determine how measures of VI affect golfing performance and whether other metrics other than VA are required.

## Key points

- Current classification systems in vision-impaired golf do not fulfil the aim to minimise the impact of vision impairment on the outcome of competition.
- An evidence-based, sport-specific classification system that reduces intentional misrepresentation is required in vision-impaired golf.
- Any new classification system developed for vision-impaired golf needs to minimise intentional misrepresentation.

## INTRODUCTION

Participation in physical activities through organised sport can provide physical and mental health benefits, particularly for individuals with disabilities such as visual impairment (VI).^1^ Being able to participate in a sporting activity can provide hope and a sense of accomplishment. With adequate support and opportunities, some individuals may even have the opportunity to compete nationally, internationally or at an elite Paralympic level. When competing with a disability, the impairment may influence the competition outcome, and therefore, creating an equitable competitive environment is a foundation of Paralympic sport.

To ensure that competitive environments are fair, athletes are evaluated to classify the severity of their impairment.^2^ The classification process determines whether an athlete is eligible to compete, and if so, which class they should compete in. Originally, athletes were classified based on the severity of their disability. Different sports, however, have specific visual demands, and so an athlete's vision profile may be more of a disadvantage in one sport than another.^3^ Taking this into account, the International Paralympic Committee have asked sport federations to develop their own sport-specific classification systems and that these should be evidence-based.^4^ For inclusion as a Paralympic sport, such sport-specific classification is now mandatory.

Sport-specific classification systems are beginning to appear.^5^ There are 11 sports currently on the Paralympic programme for athletes with a VI. Unlike physical impairment, VI is assessed whilst wearing the best vision correction (e.g., using spectacles or contact lenses). At present, the athlete with a VI needs to meet minimum impairment criteria to be eligible to compete. Once eligible, the athlete is placed into one of three classes: B1 that includes athletes with the greatest impairment, B2 representing athletes with a medium impairment and B3 being athletes with the least impairment. However, it is unknown whether this classification is optimal in different VI sports. New classification systems not adhering to this model are being trialled for shooting and are underdevelopment for football, skiing, athletics and swimming.^6–11^ Other sports such as VI golf are currently not eligible as a Paralympic sport, partly due to the lack of an evidence-based classification procedure.

Vision-impaired golf is played globally by athletes with a range of visual impairments.^12^ It is similar to sighted golf with the primary difference being that each VI golfer has a guide assisting them with aspects such as their alignment and the direction of their swing. Further modifications for those with a VI include permitting players to ground their club in circumstances that they usually would not be allowed to (i.e., when taking a shot in a bunker or sand trap). At present, eligibility to compete is based on governing body guidelines. Golfers with disabilities wishing to play in European Disabled Golf Association (EDGA) competitions can apply for a player pass called a WR4GD (World Ranking for Golfers with a Disability) pass, which allows them to collect world ranking points. Golfers are assessed to determine that their disability is severe enough to qualify for this pass.^13^ The VI requirements are currently under review at the time of writing. The International Blind Golf Association (IBGA) base eligibility on visual acuity (VA) is in the ‘better’ eye or both eyes. B1 specifies a golfer with light perception, the B2 class incorporates from B1 up to a maximum VA of 2/60 and B3 incorporates from B2 up to a maximum VA of 6/60.^14^ Generally, B4 players are not eligible to complete, although some associations have introduced a B4 category from B3 up to a maximum VA of 6/36.^15^

There are some concerns about the current classification systems used in VI golf as it is not evidence-based and uses only VA. Measures other than VA, such as visual fields^16^ or contrast sensitivity,^7^ may have a significant effect on sporting performance but are not currently part of the classification system. The relationship between level of VI and golfing performance is yet to be elucidated. Moreover, the paperwork for eligibility is submitted by the athlete after being assessed by their eye care practitioner. This will inevitably lead to a lack of standardisation in the process. There is currently no secondary confirmation of the measures by trained classifiers/assessors.

Developing such a classification system requires a systematic approach as outlined by Ravensbergen et al.^8^ The first step is identifying the aim of the classification system. How the current system is being used and whether it is robust and fair. Second, to determine which measures of visual function best identify the effects of the VI on the sport and how the unique aspects, such as the role of the guide, should be considered within the classification.

To start this process, the first step is to gather expert opinion on these questions. Such an expert consultation has been successfully undertaken by means of a Delphi review, which relies on a panel of experts to give their opinions through consecutive online surveys in a series of consultation rounds.^17^ This approach is appropriate when little is known about a research area, and therefore, expert opinions are the most reliable data available.^18^ Delphi reviews have been utilised in establishing consensus for other VI sports, such as track athletics, judo, football and swimming,^6,8,9,19^ as well as other non-VI disability sports.^20–22^

To work towards the development of a classification system for VI golfers, the objective of this study was to gather expert opinions on VI classifications in a Delphi study. This aimed to seek consensus regarding: (1) the aim of classification procedures, (2) how the current classification systems are being used, (3) which measures of visual function are required, (4) whether current procedures for classification are robust and fair, (5) whether the age that a VI was acquired should be considered in classification and (6) whether the role of guides should vary with the severity of the VI.

## METHOD

### Study design

A Delphi study was conducted in order to gather expert opinion regarding an evidence-based classification system for VI golfers. The Delphi approach is a structured method to systematically consult a panel of expert stakeholders, with the goal of reaching consensus in a relatively efficient and cost-effective way.^23^ The study was approved by the Faculty Research Ethics Panel at Anglia Ruskin University (FSE/FREP/20/988) and conformed to the tenets of the Declaration of Helsinki. The checklist of the Conducting and Reporting of Delphi Studies (CREDES) was used to report this study.^24^

### Delphi stakeholder recruitment

A panellist sample size of 20 experts was sought, based on guidelines indicating that 15–30 panellists are required for Delphi panels.^25^ Four expert groups were selected; the majority (75%) sought were VI golfers with different levels of VI, with the other 25% being guides, coaches and VI golfing officials. Golfing officials refer to individuals who facilitate, promote and manage the sport of VI golf. Eligibility criteria to be a panellist were the ability to respond online to the surveys and expertise in VI golf, defined as extensive involvement in the sport. Purposeful sampling was used to recruit internationally so that the panel represented all continents and stakeholder groups. Recruitment was through EDGA and IBGA's social media accounts and emails sent to their VI golfers. Stakeholders could ask for the survey to be translated.

One participant required surveys translated into German. This was performed using DeepL Translator,^26^ and returned survey responses were translated into English using the same software.

### Procedure

**FIGURE 1:**
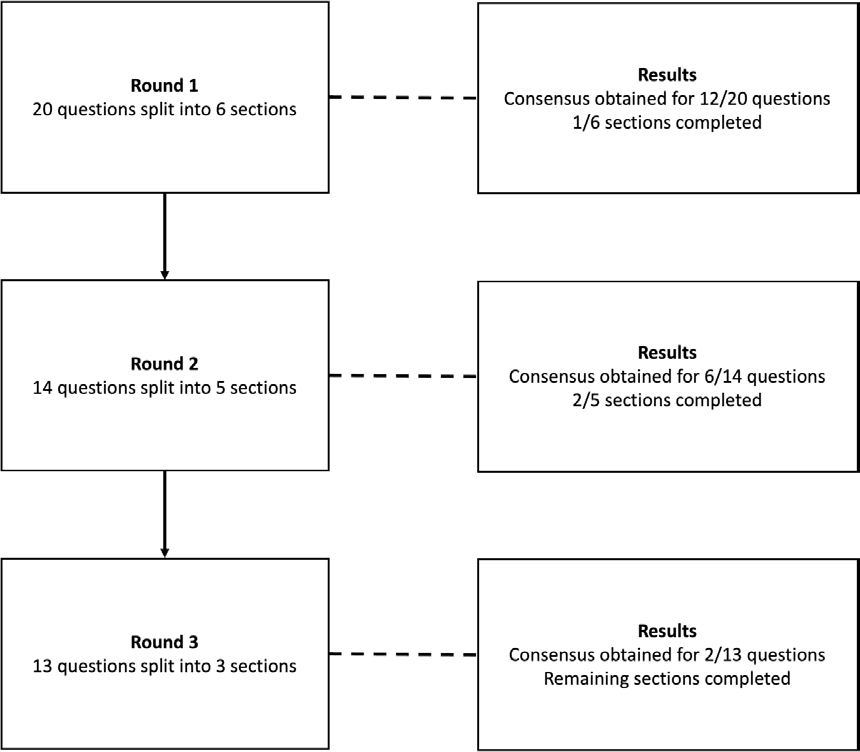
Flow diagram of the Delphi study process.

A three-round Delphi review process (Figure 1) was used to identify which areas regarding VI golf classification reached consensus. Consensus was defined as 75% or greater panellist agreement.^23,27,28^ When consensus was not achieved, suggestions made by the stakeholders were implemented, and questions were restated in subsequent rounds. Once consensus was reached on a conceptual idea, there were no follow-up questions in the subsequent rounds. As not all participants may have the expertise to answer every question, the option of not feeling qualified to answer was added to each question. Those who did not feel qualified to answer were not included when calculating consensus. Participants had the opportunity to comment on each question, allowing them to explain why they had given the answers they did. This information was used in the formation of questions in later rounds.

The survey was piloted by an advisory group of experts in the field to ensure questions were relevant to VI golf. Free-text comment boxes were used to allow for any additional feedback or thoughts on the questions asked. There were seven questions concerning demographic information and experience in VI golf at the start of the survey.

### Round 1 survey development

The first round of the Delphi study consisted of 20 questions that were split into six sections investigating: (1) the aim of classification, (2) current vision-impaired classifications, (3) measurement of VI, (4) procedures for VI classification, (5) the effect of congenital versus acquired VI and (6) the role of guides in VI golf.

### Round 2 survey development

The second Delphi survey was developed based on the results and feedback ascertained in the first survey. There were 14 questions split into five of the sections covered in the previous survey, with the questions on the effect of congenital versus acquired VI concluded. Based on the feedback from the first survey, the number of tests used to measure VI was expanded. Before each section, respondents were informed of the results in that section from the prior survey.

### Round 3 survey development

The third Delphi survey was developed based on the results and feedback in the second survey and consisted of any remaining areas where investigation was required. Two questions from the second survey were repeated due to technical errors, preventing some options being included. The third survey consisted of 13 questions split into three sections, with the sections investigating the current vision-impaired classifications and the role of guides concluded in the second survey. Written feedback was sought on what tests should be used to measure VI. Each test was investigated separately with its own free-text comment box to get feedback for each individual test, as opposed to one free-text comment box for the tests, as in the second survey.

### Survey administration

All three survey rounds were hosted online using Qualtrics (Qualtrics.com). As this software added accessibility features, it was suitable for individuals with VI. Surveys were checked by team members to ensure they worked correctly online. Panellists were given four weeks to complete and return each survey. The estimated completion time for surveys was 30 min for survey one and 25 min for surveys two and three. All participants provided electronic informed consent before completing the first survey. There was no monetary compensation or incentive offered for participation. Each survey was distributed online with each participant being emailed a link to the survey. The results of each round were analysed, and participants were informed of the results when completing the following survey.

## RESULTS

### Participants

Twenty-two participants were invited to take part in the Delphi study. Twenty individuals completed the first survey (*n* = 20), 19 completed the second, and 17 completed the third and final survey. Seventeen were men and three were women and represented multiple geographical locations. The mean duration of golf experience was 15.25 ± 9.54 years. Demographic information is presented in Table 1.

**TABLE 1:** Demographic characteristics of participants that participated in the first visual impaired golf Delphi survey

Demographics	
Sex	
Male	17
Female	3
Age (years)	
35–44	4
45–54	4
55–64	7
65+	5
Location	
Africa	3
Asia	1
Americas	4
Australasia	2
Europe	10
Role^a^	
Players	17
Coach	1
Guide	1
Officials	6
Golf experience	
<10 years	5
10–20 years	11
>20 years	4

^a^ Note that some participants had multiple roles.

The Delphi study consisted of three rounds. The results for each round are outlined in the sections below.

### Delphi survey round 1

Consensus was obtained on 12 of the 20 questions (60%) asked in the first survey (Table 2). It was agreed that the current classification system either did not fulfil or only partially fulfilled the aim of minimising the impact of VI on the outcome of competition (80% agreement). Comments suggested that certain classes were too broad and did not include other players with VI such as those with visual field defects.

**TABLE 2:** Consensus results for each of the questions asked in the first survey

Question	Level of agreement	Number of experts responding
**Section 1: Aim of Classification**		
Do you believe that the current classification system(s) for VI golf fulfils the aim to minimise the impact of VI on the outcome of competition? I believe that the current classification system(s): Totally fulfils the aim to minimize the impact of VI on the outcome of competition Partially fulfils the aim to minimize the impact of VI on the outcome of competition Does not fulfil the aim to minimize the impact of VI on the outcome of competition	15% 65% 20%	20
Which of the following options reflects your views on VI golfers competing in able sighted golf? I believe that a golfer with a VI should: Be allowed to compete in both able-sighted and VI competition Not be allowed to compete in both able-sighted and VI competition	100%* 0%	18
**Section 2: Current vision-impaired classifications**		
Do you believe that a classification system is necessary to separate eligible golfer athletes with a VI into different classes?	90%*	20
Do you believe that there should be one internationally agreed classification system in VI golf?	89%*	19
Do you believe that a classification system is necessary to separate eligible golfer athletes with a VI into different classes?	95%*	19
Do you believe the current classification system(s) may be excluding golfers whose golfing performance is affected by their VI?	63%	19
**Section 3: Measurement of VI**		
Do you believe that assessing VA provides an appropriate test to assess the impact of VI on golfing performance?	65%	17
Do you believe that the assessment of VA is the only measure of visual function that should be used for classification in VI golf?	24%*	17
What vision chart should be used to determine the VA in golfing athletes with VA?	70% Snellen, 30% LogMAR	10
Under what conditions should VA measurements be made?	Dim light conditions 0%, Normal light conditions 45%, Bright light conditions 0%, All of the above 55%	20
Do you believe that other measures of visual function should be incorporated into the classification for VI in golf?	75%*	16
Do you believe that any of the following measures of visual function should be included when determining if a golfing athlete has a VI? (You can choose more than one answer).	Glare Test, 45% Contrast Sensitivity, 60% Visual Fields, 85%* Colour Vision, 30% Motion Perception, 45% Other, 35%	20
Which specific aspects of golf (if any) do you believe would be affected in the presence of a VI? You can choose as many or as few options as you want to.	Tee-shots, 75%* Fairway Shots, 75%* Rough Shots, 85%* Chipping, 85%* Bunker Shots, 90%* Putting, 85%*	20
**Section 4: Classification procedures**		
Which of the following statements do you agree with most? Classification should be based on the results when the golfer athlete performs the vision test with:	Best eye 35%, Both eyes 65%	17
Do you believe that there is any possible scenario where a VI golfer would not be able to wear their best optical correction during competition?	46%	13
During classification, should a golfer’s visual function be assessed while wearing their best optical correction?	66%	15
Do you believe that some VI golfers are currently intentionally misrepresenting (i.e., making their VI appear better or worse than it actually is) their level of VI?	82%*	17
**Section 5: Age of VI Development**		
Do you believe that the age at which a VI is acquired should be taken into account during classification?	6%*	17
Imagine that evidence shows that the age that golfer athletes acquire their VI significantly impacts their ability to acquire skill in golf. For golfer athletes who are completely blind, do you believe that the benefits of accounting for the age that a golfer athlete acquired their impairment would outweigh the added complexity in classification?	20%*	15
**Section 6: Role of Guides**		
Should the role of a guide vary depending on the level of VI in a golfer athlete?	53%	19

*Note*: The questions that were asked, whether consensus was achieved and its value, and the number of respondents that felt qualified to answer a question are included in each row. To reach a consensus, 75% of participants would need to agree on the same answer.

Abbreviations: logMAR, logarithm of the minimum angle of resolution; VA, visual acuity; VI, Visual impairment.

* Indicates that consensus was reached.

It was agreed that other measures of visual function should be incorporated (75% agreement) in addition to VA, with visual fields achieving consensus as a measure that should be included (85% agreement). Other tests such as depth perception were suggested. To address this, the list of measures of VI was expanded and re-examined in the second survey.

Consensus was achieved on the differences in congenital and acquired VIs, with respondents agreeing that the age at which a VI is acquired should not be considered during classification (94% agreement), even when completely blind (80% agreement). Therefore, this section was concluded in the first survey.

Areas not reaching consensus included the role of guides and the use of visual correction during competition. These areas were explored in more detail in the second survey, including more opportunities for written comments to understand why these areas had differences of opinion.

### Delphi survey round 2

Consensus was obtained on six of the 14 questions (43%) asked in the second survey (Table 3). Two of the questions (related to medical assessment) in the online survey had glitches where some of the options could not be selected. As a result, these questions were repeated in the third survey. Participants were given an explanation of what each test measured during the survey to aid understanding. VA (100% agreement), visual fields (93% agreement) and depth perception (88% agreement) were deemed measures of VI that should be used during classification. To investigate the reasoning for why tests should or should not be included, each measure of VI was separated into its own question for the third survey with its own free-text comment box.

**TABLE 3:** Consensus results for each of the questions asked in the second survey

Question	Level of agreement	Number of experts responding
**Section 1: Aim of Classification**		
Please select the option you agree with the most. Golfing athletes should: Compete in classes with no golf handicap allowance Compete in classes with a golf handicap allowance in each class Compete in one overall class with a golf handicap allowance	27% 56% 17%*	18
**Section 2: Current vision-impaired classifications**		
Do you think there should be separate classifications depending on the level of competition (regional, national, international)?	17%*	18
Do you believe that golfing athletes with a VI that allows vision that is better than 6/60 to 6/36 (i.e., B4 class) have a disadvantage relative to those with no VI when playing golf?	71%	17
Should B4 golfing athletes be able to play in VI competitions?	83%*	18
Should B4 golfing athletes be able to compete in the same competitions as B1, B2 and B3 golfing athletes?	70%	18
Do you think that visual fields should be included in VI golf classifications?	86%*	14
Do you believe that there are golfers, whose golfing performance may be affected by their VI, not included in the current classification system(s)?	85%*	13
**Section 3: Measurement of VI**		
Which of the following tests do you believe should be used in the measurement of visual function when determining if a golfer has a VI?	VA, 100%* Visual field, 93%* Dynamic VA, 57% Ocular coordination, 60% Depth perception, 88%* Motion perception, 46% Contrast sensitivity, 64% Light sensitivity, 73% Colour Vision, 30%	17 17 18 17 18 18 17 18 17
**Section 4: Classification procedures**		
Who should be responsible for carrying out the measurement of visual function when determining if a golfer has a VI?	Optometrist, 53% National Association, 0% Combination of both, 47%	19
Should classification/assessment be conducted/confirmed at the venue prior to an event by an independent classifier?	50%	16
Are there any aspects of the game of golf where a golfer with a VI would not be able to wear their best optical correction during competition?	31%	16
**Section 6: Role of Guides**		
Should access to a guide vary depending on the level of VI in a golfer athlete?	84%*	19

*Note*: The questions that were asked, whether consensus was achieved and its value, and the number of respondents that felt qualified to answer a question are included in each row. To reach a consensus, 75% of participants would need to agree on the same answer.

Abbreviations: VA, visual acuity; VI, visual impairment.

* indicates that consensus was reached.

Sections two and six, covering current VI classifications and the role of guides, respectively, were concluded as these areas had been covered satisfactorily. There was agreement that there should be the same classification regardless of the level of competition (83% agreement). There was consensus that B4 athletes should be able to play in VI competition (83% agreement) and that there were currently VI golfers whose performance was affected by their impairment not included in the current classification system (85% agreement). Consensus was not obtained whether the B4 class has a disadvantage to their golfing performance relative to those with no VI (71% agreement) or whether B4 golfing athletes should be able to play in the same competitions as VI golfers with more severe impairments (70% agreement). Eighty-six per cent of participants agreed that visual fields should be included in VI golf classifications. Access to a guide should not vary depending on the level of VI (84% agreement).

In section 1, there was agreement that the class system should continue in one form or another (83% agreement). Based on the results and feedback, a further question on the role of a golf handicap system being incorporated within each class was included in the third survey.

### Delphi survey round 3

Consensus was obtained on two of the 13 questions asked in the third survey (Table 4). In this survey, questions about vision assessments were asked again but this time included a free-text box. This was to better understand why participants were choosing why a test should be included or not. The use of visual field assessment (61% agreement) and depth perception (63% agreement) to measure VI no longer reached consensus, with VA (100% agreement) the only measure to do so. Colour vision reached consensus not to be used to assess VI (15% agreement). For visual fields, there were opposing arguments whether they should be used, with some respondents saying that central vision was more important than peripheral vision in the sport. There was consensus that a medical assessment of the eyes should be part of the classification process to reduce or minimise intentional misrepresentation of VI (94% agreement). However, there was no agreement on how this should be funded.

**TABLE 4:** Consensus results for each of the questions asked in the third survey

Question	Level of agreement	Experts
**Section 1: Aim of Classification**		
Do you think there should be a golf handicap incorporated within each classification class?	67%	17
**Section 3: Measurement of VI**		
Should VA (a measure of the sharpness/clarity of vision) be used as a measure of visual function when determining if a golfer has a VI?	100%*	15
Should visual fields (a measure of the area of peripheral vision with which an individual can see (i.e., without moving their eyes) be used as a measure of visual function when determining if a golfer has a VI?	61%	13
Should depth perception (the ability to perceive the world in three dimensions, e.g., to estimate the distance to an object) be used as a measure of visual function when determining if a golfer has a VI?	63%	16
Should light sensitivity (the impact of bright lights on the ability to see clearly) be used as a measure of visual function when determining if a golfer has a VI?	33%	16
Should contrast sensitivity (the ability to distinguish objects from a background) be used as a measure of visual function when determining if a golfer has a VI?	50%	16
Should ocular coordination (the ability of both eyes to move together in tandem) be used as a measure of visual function when determining if a golfer has a VI?	33%	12
Should dynamic VA (a measure of the sharpness/clarity of vision when observing a moving target) be used as a measure of visual function when determining if a golfer has a VI?	69%	13
Should colour vision (the ability to distinguish different colours from another) be used as a measure of visual function when determining if a golfer has a VI? Please explain your answer in the comment box.	15%*	13
Should motion perception (the ability to estimate the speed and the direction of a moving object) be used as a measure of visual function when determining if a golfer has a VI?	40%	15
**Section 4: Classification procedures**		
Should VI golfing athletes only compete in the class they are eligible for with their best corrected VA (i.e. with their optimum spectacles /prescription sunglasses / contact lenses)? Please explain your answer in the comment box.	64%	14
Should there be a medical assessment of the eyes as part of classification in order to reduce or minimise intentional misrepresentation of VI? Please explain your answer in the comment box.	94%*	17
If classification requires medical assessment, how should it be funded?	Golfing Athlete, 59% Organising Body, 29% National Federations, 12%	16

*Note*: The questions that were asked whether consensus was achieved and its value and the number of respondents that felt qualified to answer a question are included in each row. To reach a consensus, 75% of participants would need to agree on the same answer.

Abbreviations: VA, visual acuity; VI, visual impairment.

* indicates that consensus was reached.

## DISCUSSION

A three-round Delphi study was conducted with 20 stakeholders in VI golf from across the globe covering several areas affecting the classification of the sport. The purpose was to gather expert opinions regarding the current classification system, and what needs to be considered in the development of an evidence-based classification system for golfers with a VI. There were several sections as discussed below.

### Aim of classification—What system is best (handicap/no handicap)—What happens with B4s?

The stakeholders indicated that the current classification system did not minimise the impact of VI on golfing performance and hence is not fit for purpose. Similar findings have been noted recently in VI football, swimming, judo and athletics, suggesting that many classification systems do not meet their aim in VI sport.^6,8,9,19^ Thus, there is a need to consider which measures of visual function are appropriate to reflect the impact of VI on golfing performance and hence should be included in a classification system.

### Current system

The panel suggested the need for one internationally agreed classification system that could be used at all levels of the sport. One of the key areas of debate in this study was the minimum impairment criteria (MIC) required to compete in VI golf to make it a fair system. This refers to the minimum level of VI that affects golfing performance. The second key area was a desire to grow the sport. It was identified that as VIs impact golfing performance, the inclusion criteria were too stringent, allowing only golfers with VI in the B1 to B3 classes to compete. Concerns were also present that widening the inclusion criteria would allow athletes to compete whose VI did not affect their performance, as has been the case for footballers and judokas.^9,19^ To achieve fair inclusion criteria, it is necessary to identify at what level VI starts to affect performance. Such studies have been undertaken for other VI sports such as shooting, Nordic and alpine skiing.^7,10,11^ While there has been work that identified 10 D of defocus can impact putting,^29,30^ further research is necessary. In cricket, batting was found to be affected by 3 D of defocus, which suggests that the severity of defocus can vary before affecting individual techniques in sport.^31^

### Measurement of VI

The panellists suggested that VA should not be the only measure of visual function included in the classification. Although full consensus was not reached, visual fields were consistently mentioned in comments as an area that should be considered in the assessment of VI. Depth perception was also suggested to be important. Historically, the classification system for Paralympic sports has incorporated visual fields into their B2 and B3 classes, including athletes with a visual field of less than 10° and 40°, respectively. Not all sports allowed the B2 and B3 classes, with only B1 class allowed to compete in 5-a-side football, for example, whilst others, such as athletics and rowing, do allow the B2 and B3 classes to compete at a Paralympic level.^32^ More recently, VI sport is going through a period of transition. Sports such as judo^33^ and shooting^34^ have changed from the traditional VI categories to more sports-specific systems. Classification for VI shooting^6,34^ now includes measures of contrast sensitivity, not previously considered in its classification systems. The lack of visual field testing within a classification system will exclude golfers with progressive conditions such as retinitis pigmentosa or glaucoma. These conditions generally affect peripheral vision much earlier and may not affect VA until later in the condition when the person only has a very restricted visual field. Currently, a golfer can have a moderate-to-severe visual field defect but not be eligible to compete in a VI golf competition. Research is required to identify the impact of visual field loss (central and peripheral) on golfing performance.

### Procedures for VI classification—Including intentional misrepresentation

The panel indicated that the current classification process does not adequately prevent intentional misinterpretation due to the golfer submitting their own evidence via an online system. Forty-seven per cent of the panel indicated that national associations should undertake the testing for classification, whereas others felt that independent optometrists could do the testing. The location of classification was also mentioned as to whether it should be at the sporting event or from local eyecare practitioners. Although testing at centralised venues could improve standardisation, it may affect accessibility for those unable to travel to these centres.

The panel indicated that intentional misrepresentation of VI is common and that steps should be taken to minimise it. Undertaking a medical assessment was identified as important to include in an attempt to reduce or minimise intentional misrepresentation of VI, in keeping with other Paralympic VI sports. This is one of the challenges when classifying athletes in VI sport, particularly with the increased profile and recognition associated with the Paralympic games.^16^ In terms of limiting the issue, the use of more objective rather than subjective testing was proposed. Employing methods such as electroretinography would serve this purpose but are costly, and availability is less widespread than other methods of vision screening. Any adaptations to the current or future classification procedures need to be mindful of potential intentional misrepresentation of VI, while ensuring that it is accessible to those that require it.

### Age of VI development

The panel agreed that the age at which a VI developed, and whether it is congenital or acquired, should not be considered during classification regardless of the severity. The same conclusion was reached for VI swimming^8^ and VI athletics, although stakeholders thought that athletes born with a VI had a disadvantage over those who acquired a VI later on in life.^6^

### Role of guide

Guides are essential in assisting the VI golfer. It was unclear whether guide involvement should increase with the level of VI. This interaction should be explored further.

## CONCLUSION

This study has certain limitations. Although the study sought to include a diverse range of panellists, it was not possible to represent the full range of golfing experience, handicap and type of VI. Furthermore, panellist's views were all counted equally, and do not consider the panel member's experience within VI golf. There were also fewer female panellists (although this is similar to VI golf in general where far fewer females participate).

In regard to developing an evidence-based system, stakeholders indicated that having one internationally agreed classification system in VI golf is required. Any future research into the establishment of such a system will require a unified approach from associations and rule-making bodies such as EDGA, IBGA, the Royal and Ancient Golf Club of St Andrews and the United States Golf Association. It is also important to note that golf for people with disabilities incorporates players with varying disabilities, the impact of which need to be considered.

In conclusion, this Delphi study demonstrates that the current classification systems do not adequately minimise the impact of VI on the outcome of competition. There is a clear need for an evidence-based, sport-specific, classification system that minimises intentional misrepresentation. The MIC for VI golf needs to be determined and, once established, a classification system developed. Qualitative research should also be used to investigate the barriers and facilitators in VI Golf.
